# Assessing the Body Composition of “Picky Eaters” Using Body Impedance Analysis: An Experience From a Tertiary Care Center

**DOI:** 10.7759/cureus.60538

**Published:** 2024-05-18

**Authors:** Rola Sleiman, Wessam Abdelkader, Dana AlTannir

**Affiliations:** 1 Pediatrics, Dr. Sulaiman Al Habib Medical Group, Riyadh, SAU; 2 Pediatric Medicine, Alfaisal University College of Medicine, Riyadh, SAU

**Keywords:** weight, height, appetite, bia, body composition, picky eaters, children

## Abstract

Background: Picky eating might be associated with a higher risk of being underweight and poor growth. This study aimed to investigate if picky eaters aged between three and 12 years showed differences in height, weight, and body composition compared to their non-picky peers using a body impedance analysis (BIA).

Methods: This cross-sectional study was conducted between March 1, 2022, and July 31, 2022, on children aged three to 12 years who presented to the outpatient pediatric clinics at Al Habib Ar Rayyan in Riyadh, Saudi Arabia. Body composition was measured using BIA after manually inserting the height, gender, and age into the machine, where body mass index (BMI), fat mass, and skeletal muscle mass were recorded. Participants were classified as under/normal/over for each body composition measurement.

Results: A total of 2234 children were entered into the final data analysis. Our analysis showed that 1917 (85.8%) were Saudis and 1117 (50%) were males. The mean age of participants was 6.08±2.01 years and 1151 (51.5%) were in the pre-school age. The reported prevalence of picky eaters was 1684 (75.4%), of whom, 606 (27.0%) were selective eaters, 365 (16.2%) were low appetite eaters, and 723 (32.2%) were both selective and low appetite eaters. Being underweight was significantly more common among the picky eaters 487 (28.9%) compared to those non-picky eaters 55 (10.0%) (p<0.001). Significantly, 1280 (76%) picky eaters had below-average skeletal muscle mass compared to 151 non-picky eaters (27.5%) (p<0.001). The low appetite picky eater group had more under skeletal muscle mass children 277 (75.9%) compared to the selective picky eater group 412 (68.0%) (p=0.009). Additionally, the low appetite group possessed lower muscle ratios (p=0.012) and were more underweight than the selective group (p<0.001). Furthermore, the low appetite group showed a higher percentage of children below the 3rd percentile in the height for age category (p=0.003) compared to the selective group.

Conclusion: This study is the first of its kind in Saudi and globally to evaluate body composition using BIA among children. The study showed that picky eating is more associated with underweight children and low muscular mass. Despite the normal BMI, height, and weight of a picky eater, their skeletal muscle mass might be less than average, which could be associated with an increased risk of morbidity.

## Introduction

Picky eating is a common behavior observed in childhood [1-3]. Picky eating is defined as the rejection of a wide range of familiar and unfamiliar foods, leading to limited and insufficient food intake [2,4]. However, there is currently no universally agreed-upon medical definition of picky eating, resulting in varying definitions across the literature [5]. This has led to a wide range of reported prevalence rates of picky eating in pediatrics across different countries, ranging from 6.4% to 54% [6-8]. While several studies have examined the prevalence of picky eating in childhood, few have investigated its impact on body composition and development [1]. Growth and body composition are determined by weight, height, body mass index (BMI), fat mass, and skeletal muscle mass [9]. These studies have produced conflicting results, with some suggesting that constant picky eating behavior in early childhood leads to lower height and weight [6,10,11]. Dual-energy x-ray absorptiometry (DEXA) is the primary technique used to evaluate body composition, measuring bone density, fat, free fat mass, and lean mass index [12]. However, no studies have assessed the body composition of picky eaters using body impedance analysis (BIA), which is a non-invasive and quick method for measuring body composition and is thus more applicable to large-scale studies [13]. According to our literature search, this study is the first of its kind in Saudi Arabia and globally to use BIA to evaluate body composition among pediatric picky eaters, unlike the conventional methods of measuring weight, height, and x-ray absorptiometry [7,8]. This study aimed to assess the prevalence of picky eaters aged between three and 12 years old using BIA to identify their body composition characteristics in a tertiary care center in Riyadh.

## Materials and methods

Study design, setting, sample, and sampling technique

This study was an analytical observational cross-sectional study conducted on children between March 1, 2022, and July 31, 2022. This study included children aged three to 12 years who live in the Riyadh metropolitan area and presented to the outpatient pediatric clinics at Al Habib Ar Rayyan in Riyadh. The willingness to participate in the study was documented by written consent of the parent/guardian. Children under the age of three years, above the age of 12 years, having chronic illnesses affecting eating habits and growth status, mental disorders, genetic diseases, psychiatric illness, gastroesophageal reflux disease, esophagitis, food allergies, lactose intolerance, known disability, prematurity, low birth weight (<2500 g), or in-patients were all excluded from this study.

Data collection procedure

Data for each child was composed of two section parts. Section one included sociodemographics such as nationality (Saudi or non-Saudi), gender (male or female), and age measured as a continuous variable and then divided into the following two categories: preschoolers from three to six years and school children from seven to 12 years.

The second section measured the body compositions using the impedance machine - Inbody 270 (Los Angeles, CA: Biospace), the height and weight using a wall scale, as a part of the routine tests upon clinic visitation. The consultant manually plugged in the gender, age, and height into the Inbody 270 machine and screened for inclusion/exclusion criteria. The machine measured: weight, height, body mass index (BMI), fat mass, and muscle mass. The Inbody 270 machine was validated for use among the pediatric population from the age of three years onwards [14,15]. The results sheet of the Inbody machine was then scanned into the electronic file of each patient, and a pediatrician analyzed the results produced by the machine. Height was measured using a wall scale after the children took off their shoes, and it was taken twice for accuracy. The Inbody 270 machine uses the latest CDC growth chart; thus, the results are comparable to the average measurement for the age percentile set by the CDC growth charts [16]. The term "under" was used to indicate that the child has less than the average measurement (fat, muscle, height, BMI, and weight) for the age percentile, "normal" was used to indicate that the child has average measurement (fat, muscle, height, BMI, and weight), while "over" was used to indicate that the child has higher than the average measurement for age percentile (fat, muscle, height, BMI, and weight). Height under the third percentile and under skeletal mass were recorded using a binary value (yes, no).

Picky eating and its subtypes were identified using two simple questions. The first question assessed whether the child was considered a picky eater by asking the parent, in their language, Arabic or English, the question: "Is your child a picky eater?" If the parent answered with a yes, then the child was classified as a picky eater and a follow-up question was asked to assess the selectivity and appetite of the child. This was done by asking the parent: "Is he selective, or is he eating small portions of food?"

Selective means they only eat certain kinds of food while having a low appetite is equivalent to eating small portions of food. Selective eating involves consuming only specific types of food that an individual deems safe or suitable, steering clear of those with particular tastes, textures, or colors. On the other hand, having a low appetite entails not experiencing hunger, lacking the desire to eat, or consuming minimal amounts of food. This may be due to feeling satiated or disliking the taste, appearance, or aroma of certain foods (food aversion) [2,4]. Thus, the picky eaters were classified into the following three subgroups: selective, low appetite, or both (selective and low appetite).

Data analysis

We used SPSS version 22 (Armonk, NY: IBM Corp.), released in 2012, to analyze the data. Categorical variables were summarized using descriptive statistics as frequencies, percentages, and continuous variables as means and standard deviation. The chi-square test was used to compare categorical variables and the independent sample t-test was used to compare two means. We carried out a binary logistic regression to determine whether the picky eaters have significant changes in body composition compared to non-picky eaters, and to identify predictors of picky eating and predictors of low appetite picky eaters. The variables included in the binary logistic regression model were selected based on statistical significance variables with p-values of <0.025 on the univariable analysis were included in the model. P-values of less than 0.05 were considered significant.

Ethical considerations

Ethical approval for this study was obtained from the Institutional Review Board (IRB) of Dr. Sulaiman Al Habib Research Center, Riyadh, Saudi Arabia. The willingness to participate in the study was documented by signing the written informed consent of the parent/guardian and oral assent obtained from children above six years. The identity of the participants was kept confidential and maintained all the time. All data were stored in a secure place that was only accessed by the researcher.

The conduct of clinical research at our institution is governed by the highest ethical codes and the National Committee of Bioethics in Saudi Arabia. It adheres to global standards for ethical research involving humans, as outlined in the Belmont Report, the Helsinki Declaration and its revisions, and the International Ethical Guidelines for Health-Related Research Involving Humans.

## Results

Table 1 showed that a total of 2234 children were entered into the final data analysis, of whom 1684 (75.4%) were picky eaters and 550 (24.6%) were normal eaters. Additionally, 650 (29.1%) and 660 (29.5%) of the children fell below the 50th percentile of their age group for height and weight, respectively. Furthermore, 542 (24.3%) children were underweight and 1431 (64.1%) were under-muscle (Table 1).

**Table 1:** Participants’ demographics and body composition characteristics.

Characteristics	Number	Percentage (%)
Nationality		
Saudi	1917	85.8
Non-Saudi	317	14.2
Gender		
Male	1117	50
Female	1117	50
Age (years)		
Mean±SD	6.08±2.01	-
Pre-school	1151	51.5
School-age	1083	48.5
Weight for age percentiles		
<3%	41	1.8
3% to <50%	660	29.5
≥50% to 97%	1355	60.7
>97%	178	8
Height for age percentiles		
<3%	47	2.1
3% to <50%	650	29.1
≥50% to 97%	1509	67.5
>97%	28	1.3
Fat		
Under fat	189	8.5
Normal fat	1165	52.1
Over fat	880	39.4
Muscle		
Under muscle	1431	64.1
Normal	771	34.5
Over muscle	32	1.4
BMI		
Underweight	542	24.3
Normal	956	42.8
Overweight	736	32.9
Height under 3 percentile		
Yes	47	2.1
No	2187	97.9
Under skeletal muscle mass		
Yes	1431	64.1
No	803	35.9
Picky or normal eater		
Picky eater	1684	75.4
Normal eater	550	24.6
Types of picky eater		
Selective	606	27.0
Low appetite	365	16.2
Selective and low appetite	723	32.2

Table 2 showed that 1479 (87.8%) picky eaters were Saudis with a mean age of 6.06±2.01 years. About 38 (2.3%) picky children fell below the third percentile of weight for age percentiles, while 62 (3.7%) lay within the higher weight for age percentiles, with a statistically significant difference in gender between the picky and non-picky eaters, where picky eaters were more commonly females. Moreover, being underweight was significantly more common among the picky eaters, 487 (28.9%), than among the non-picky eaters, 55 (10.0%). Additionally, 1280 (76%) picky eaters had below-average skeletal muscle mass, while only 151 (27.5%) of non-picky eaters had below-average skeletal muscle (Table 2).

**Table 2:** Comparison of picky and non-picky eaters in relation to their demographics and body composition characteristics.

Characteristics	Is your child a picky eater?		p-Value
	Yes=1684 (75.4%)	No=550 (24.6%)	
Nationality			
Saudi	1479 (87.8%)	438 (79.6%)	<0.001
Non-Saudi	205 (12.2%)	112 (20.4%)	
Sex			
Male	807 (47.9%)	310 (56.4%)	<0.001
Female	877 (52.1%)	240 (43.6%)	
Age (mean±SD) years	6.06±2.01	6.15±2.01	0.396
Pre-school (3-6)	923 (54.8%)	228 (41.5%)	<0.001
School-age (7-12)	761 (45.2%)	322 (58.5%)	
Weight for age percentiles			
<3%	38 (2.3%)	3 (0.5%)	0.009
3% to <50%	596 (35.4%)	64 (11.6%)	<0.001
≥50% to 97%	1367 (58.7%)	367 (66.7%)	<0.001
>97%	62 (3.7%)	116 (21.1%)	<0.001
Height for age percentiles			
<3%	39 (2.3%)	8 (1.5%)	0.222
3%-<50%	534 (31.8%)	116 (21.1%)	<0.001
≥50%-97%	1179 (65.1%)	412 (74.9%)	<0.001
>97%	14 (0.8%)	14 (2.5%)	0.002
Fat			
Over fat	542 (32.2%)	338 (61.5%)	<0.001
Normal fat	982 (58.3%)	183 (33.3%)	<0.001
Under fat	160 (9.5%)	29 (5.3%)	0.002
Muscle			
Over muscle	12 (0.7%)	20 (3.6%)	<0.001
Normal muscle	392 (23.3%)	379 (68.9%)	<0.001
Under muscle	1280 (76.0%)	151 (27.5%)	<0.001
BMI (kg/m^2^)			
Underweight	487 (28.9%)	55 (10.0%)	<0.001
Normal	767 (45.5%)	189 (34.4%)	<0.001
Overweight	430 (25.5%)	306 (55.6%)	<0.001
Height under 3 percentile			
Yes	37 (2.2%)	8 (1.5%)	0.282
No	1647 (97.8%)	542 (98.5%)	0.282
Under skeletal muscle mass			
Yes	1280 (76.0%)	151 (27.5%)	<0.001
No	404 (24.0%)	399 (72.5%)	<0.001

The majority of selective 535 (88.3%) and low appetite 298 (81.6%) eaters were Saudis, with a mean age of 6.12±2.01 and 5.98±2.01, respectively. A statistically significant difference (p<0.001) was found between the two groups in weight for age percentiles. Forty-six (12.6%) low appetite picky eaters possessed significantly lower fat in comparison to the 21 (3.5%) seen in selective picky children (p<0.001). The low appetite group possessed significantly lower muscle ratios 277 (75.9%) vs 412 (68.0%) (p=0.012) (Table 3).

**Table 3:** Comparison of selective and low-appetite eaters in relation to their demographics.

Picky eaters			
Characteristics	Selective (n=606)	Low appetite (n=365)	p-Value
Nationality			
Saudi	535 (88.3%)	298 (81.6%)	0.006
Non-Saudi	71 (11.7%)	67 (18.4%)	
Age (mean±SD)	6.12±2.01	5.98±2.01	0.319
Pre-school	320 (52.8%)	205 (56.2%)	
School-age	286 (47.2%)	160 (43.8%)	
Sex			
Male	293 (48.3%)	188 (51.5%)	0.354
Female	313 (51.7%)	177 (48.5%)	
Weight for age percentiles			
<3%	9 (1.5%)	12 (3.3%)	<0.001
3% to <50%	111 (18.3%)	144 (39.4%)	
≥50% to 97%	440 (72.6%)	204 (55.9%)	
>97%	46 (7.6%)	5 (1.4%)	
Height for age percentiles			
<3%	7 (1.2%)	14 (3.8%)	0.003
3% to <50%	149 (24.6%)	116 (31.8%)	
≥50% to 97%	442(73%)	234 (64.1%)	
>97%	8 (1.3%)	1 (0.3%)	
Fat			
Over fat	317 (52.3%)	78 (21.4%)	<0.001
Normal fat	268 (44.2%)	241 (66.0%)	
Under fat	21 (3.5%)	46 (12.6%)	
Muscle			
Over muscle	9 (1.5%)	1 (0.3%)	0.012
Normal muscle	185 (30.5%)	87 (23.8%)	
Under muscle	412 (68.0%)	277 (75.9%)	
BMI (kg/m^2^)			
Underweight	111 (18.3%)	117 (32.1%)	<0.001
Normal	266 (43.9%)	174 (47.7%)	
Overweight	229 (37.8%)	74 (20.3%)	
Height under 3 percentile			
Yes	6 (1.0%)	14 (3.8%)	0.004
No	600 (99.0%)	351 (96.2%)	
Under skeletal muscle mass			
Yes	412 (68.0%)	277 (75.9%)	0.009
No	194 (32.0%)	88 (24.1%)	

Furthermore, the low appetite group had significantly more underweight children than the selective group 117 (32.1%) vs 111 (18.3%) (p<0.001). Similarly, height for age under the third percentile and under skeletal muscle mass were significantly more prevalent in the low appetite group (Table 3).

The binary logistic regression model showed that none of the independent variables were significant predictors in picky eating behavior children. However, the regression analysis showed that children with under fat were 0.122 times more likely to exhibit low appetite behaviors than selective behaviors (p<0.001). Similarly, height under the third percentile was about four times more likely in low appetite picky eater behavior (p=0.004) (Table 3).

## Discussion

The prevalence of picky eaters in this study was 1684 (75.4%), of whom 1280 (76%) had less than average skeletal muscle mass, unrelated to BMI. Decreased fat mass and height less than the third percentile are two significant independent variables associated with low appetite picky eaters.

This upper limit of the global reported prevalence could be attributed to Saudi Arabia, situated in the Eastern Mediterranean region, which stands as one of the world's rapidly expanding economies [14,17-19]. The surge in household income at the onset of the 21st century led to a swift nutrition shift, marked by a rise in the consumption of high-sugar, high-fat foods. This shift has been linked to a significant burden of obesity, food allergies, and non-communicable diseases [17]. Our prevalence is consistent with two previously conducted studies, one reported a prevalence of 59.3% in China [20], and the other by Kutbi et al. reported 89.8% in a study conducted on Saudi children [21].

In a recent study conducted in Saudi Arabia, Kutbi reported that children who display picky eating behaviors tend to have lower consumption of fruits, vegetables, and protein compared to non-picky eaters, and tend to consume higher levels of trans fat [18]. This increased intake of trans fatty acids may indicate a preference for fried or fast foods, as well as baked goods like cakes, cookies, crackers, and margarines [17,18,22,23]. In addition, Lazzeri et al. reported micronutrient inadequacies in Saudi children, with suboptimal intakes of essential nutrients including fiber, calcium, and phosphorus [24]. Therefore, knowing the unhealthy Saudi eating habits and sedentary lifestyle, the decreased skeletal muscle mass is an indicator that the selectivity of food among our study group is that of true picky eaters, and in turn a good indicator of the high reported prevalence by the parents.

In accordance with our study, Taylor et al. were the first to report suggestion of a lower lean mass index (LMI) in very picky eaters in both genders after conducting a detailed longitudinal study on the body composition of picky eaters using DEXA; however, after the adjusted regression models, Taylor et al. reported a lower LMI in boys only [11]. In addition, de Barse et al. reported lower fat-free mass in picky vs non-picky eaters at six years of age. Decreased skeletal muscle mass among children raises concerns about future metabolic, cardiovascular, and physical activity issues and is therefore associated with higher morbidity and mortality rates [19].

Several studies have reported that picky eaters are more likely to be underweight when compared to non-picky eaters [25-27]. In alignment with our study, Hanapi et al. reported that picky eaters had significantly lower weight and weight for age z scores than non-picky eaters [28]. In addition, Dubois et al. showed that picky eaters were twice as likely to be underweight when compared to non-picky-eating children by the age of 4.5 years [25]. Furthermore, Taylor et al. showed that the weight of picky eaters was lower by about 15 centile points than the weight of non-picky eaters [11].

In contrast to our findings, a few studies reported no difference in growth and body weight between picky and non-picky eaters [29,30]. Mok et al. reported that picky eaters were more likely to have a normal weight based on weight for age, height for age, and BMI for age compared with children who were not picky eaters [29]. Additionally, Berger et al. showed that persistent picky eaters were within the normal weight range for their age [30].

Our study revealed that low-appetite picky eaters had a significant decrease in fat levels and skeletal muscle ratio when compared to selective picky eaters. In addition, the low appetite group had significantly more underweight children than the selective group. This indicates that the low appetite picky eaters are not consuming their required daily nutrient intake both in amount and quality. Whereas the selective group might consume high-energy unhealthy food that contributes to their weight gain, thus making them less underweight than the low appetite group.

Strengths and limitations

This study is the first worldwide to evaluate the body composition of children using BIA; however, it has some limitations. First, this study design was cross-sectional, completed at a one-time point, and the self-reporting by parents can introduce a recall bias. Second, the convenient sampling method might introduce selection bias which could influence the results of this study. In addition, we did not use the structured validated Children's Eating Behavior Questionnaire (CEBQ), nor did we specify certain criteria of food types or record the dietary intake to identify picky eaters, rather, we used two close-ended subjective questions to identify picky eaters in this study, this may lead to information bias. Furthermore, the CDC growth charts were used as the reference in this study, so using the Saudi growth charts could have different implications.

Therefore, a large-scale school-based survey should be carried out to determine the prevalence of decreased skeletal muscle mass in children using a validated questionnaire and daily dietary intake records, in addition to studying the effect of cultural food habits, puberty, and parental selectivity on eating behaviors.

## Conclusions

This study is the first of its kind to evaluate body composition using BIA among pediatric picky eaters. The study showed that picky eating is associated with underweight children and low muscular mass. Low appetite picky eaters showed a significant decrease in fat levels and skeletal muscle ratio in comparison to selective picky eaters. In addition, the low appetite group had significantly more underweight children than the selective group. Even though picky eaters can have a normal BMI, height, and weight measures, their skeletal muscle mass might be less than the average. This raises concern for the pediatric population’s well-being since decreased skeletal muscle mass among children is associated with an increased risk of morbidity.
